# Simplified geometric representations of protein structures identify complementary interaction interfaces

**DOI:** 10.1002/prot.26020

**Published:** 2020-11-11

**Authors:** Caitlyn L. McCafferty, Edward M. Marcotte, David W. Taylor

**Affiliations:** ^1^ Department of Molecular Biosciences University of Texas at Austin Austin Texas USA; ^2^ Center for Systems and Synthetic Biology University of Texas at Austin Austin Texas USA; ^3^ Institute for Cellular and Molecular Biology University of Texas at Austin Austin Texas USA; ^4^ LIVESTRONG Cancer Institutes Dell Medical School Austin Texas USA

**Keywords:** computational biology, interaction interfaces, protein structure

## Abstract

Protein‐protein interactions are critical to protein function, but three‐dimensional (3D) arrangements of interacting proteins have proven hard to predict, even given the identities and 3D structures of the interacting partners. Specifically, identifying the relevant pairwise interaction surfaces remains difficult, often relying on shape complementarity with molecular docking while accounting for molecular motions to optimize rigid 3D translations and rotations. However, such approaches can be computationally expensive, and faster, less accurate approximations may prove useful for large‐scale prediction and assembly of 3D structures of multi‐protein complexes. We asked if a reduced representation of protein geometry retains enough information about molecular properties to predict pairwise protein interaction interfaces that are tolerant of limited structural rearrangements. Here, we describe a reduced representation of 3D protein accessible surfaces on which molecular properties such as charge, hydrophobicity, and evolutionary rate can be easily mapped, implemented in the MorphProt package. Pairs of surfaces are compared to rapidly assess partner‐specific potential surface complementarity. On two available benchmarks of 185 overall known protein complexes, we observe predictions comparable to other structure‐based tools at correctly identifying protein interaction surfaces. Furthermore, we examined the effect of molecular motion through normal mode simulation on a benchmark receptor‐ligand pair and observed no marked loss of predictive accuracy for distortions of up to 6 Å Cα‐RMSD. Thus, a shape reduction of protein surfaces retains considerable information about surface complementarity, offers enhanced speed of comparison relative to more complex geometric representations, and exhibits tolerance to conformational changes.

## 1 INTRODUCTION

Proteins often assemble into multi‐protein complexes as their native forms, mediated by pairwise (or higher‐order) protein‐protein interactions. Knowledge of the participating protein‐protein interfaces involved in forming these complexes is thus critical for understanding and perturbing protein function in a cellular context. Most of our understanding about the contact surfaces by which proteins interact has been from direct experimental determination using techniques such as X‐ray crystallography and electron microscopy,^1^, ^2^ but these methods remain costly and laborious. Other, more indirect experimental techniques, including mutagenesis,^3^, ^4^ mass spectrometry,^5^ and cross‐linking analysis,^6^ can also illuminate the specific residues that participate in these interaction interfaces. These techniques give partial information about the three‐dimensional (3D) assembly of complexes, and new integrative computational modeling strategies are increasingly able to consider such data as distance restraints to infer 3D structures.^7^, ^8^, ^9^, ^10^ To complement experimentally led approaches, there has also been a strong push to develop better computational approaches for predicting protein interaction interfaces directly from protein amino acid sequences and 3D structures.

Importantly, the prediction of protein‐protein interaction interfaces is of substantially lower computational complexity than the problem of predicting or folding a 3D protein structure based on its linear amino acid sequence, as interface predictions (eg, by molecular docking) are limited to 6° of rotational and translation freedom and a sampling of accompanying intramolecular motions that might occur upon binding.^11^ Ideally, successful interface predictors would go beyond predicting pairwise interactions and be useful to assemble large molecular machines from individual subunits.

Such predictions are complicated by the fact that protein‐protein interactions may take quite different forms, and interactions can be categorized in various ways, including obligate and non‐obligate, permanent and transient, and strong and weak.^12^ Obligate complexes consist of proteins that are not stable on their own and depend on cooperative folding between the subunits, while non‐obligate complexes form from proteins that fold alone and take part in transient or permanent protein interactions. Transient interactions can be further divided into strong and weak interactions. Several studies have determined trends in residues that form protein interfaces. For example, transient interactions have been observed to have similar proportions of hydrophobic residues on both the interaction interface and the remaining surface of the protein. However, because these interfaces are rich in water molecules,^13^ there tend to be a larger number of polar residues along the interface.^14^ Additionally, many of the forces driving these interactions derive from weak electrostatic charge.^15^ Furthermore, all of these noncovalent interactions would benefit from a calculation of the binding affinity.^16^ Thus, computational approaches face a significant challenge in predicting contact interfaces that may vary significantly based on the relevant class of protein‐protein interaction for any particular interface.

Computational approaches for determining how proteins interact include predictions of interaction interfaces or docking of protein structures, where the former informs the latter. It has been shown that knowledge of an interaction interface can greatly improve the prediction of the conformation of the proteins that are interacting.^17^ Interface predictors may be divided into two groups: intrinsic‐ and template‐based approaches.^18^ Intrinsic‐based approaches focus on features within the protein sequence or the protein structure. Template‐based approaches search through databases of protein complexes with known structures and use these interfaces to make predictions.^19^ However, the latter approach requires prior structural information for the protein(s) of interest. Intrinsic‐based approaches take either sequence information or structural information as the input of the predictor. Enhancing intrinsic‐based approaches may be challenging, as a review of previous literature found that the addition of more features does not improve predictions.^18^

Sequence‐based predictors utilize protein sequence information to either feed different amino acid properties into a machine learning classifier or sequence alignment tools. Sequence alignment methods assume that proteins of similar sequences have similar binding partners and therefore binding sites.^19^ Many machine learning techniques focus on features of neighboring residues, where the size of the window of residues ranges from 9 to 21 amino acids.^19^ However, proximity in sequence does not necessarily reflect proximity in structure, highlighting the benefits of incorporating structural information into the interface predictions. Some techniques have taken an intermediate approach where the proteins are represented by a network where individual nodes represent residues and residue properties, while edges represent structural information providing some spatial resolution.^20^, ^21^

Structure‐based predictors utilize structural information from either experimental data or homology modeling as a constraint in formulating their prediction. Previous studies showed that the quality of the prediction is dependent on the quality of the structure and that homology models produce less accurate predictions.^19^ One structural approach involves dividing a protein surface into patches and using these patches to predict interaction sites. Patches are defined as either the *n* closest residues where *n* depends on the size of the protein or a set size for all proteins.^22^, ^23^ For these methods, patch size is predetermined and uniform, causing problems for predicting interfaces of proteins with multiple binding partners or if the defined surface patch does not accurately reflect the size of the true interface.^22^ Many predictors ignore the binding partner; however, utilizing the binding partner has been shown to improve predictions.^18^

Partner‐specific interface predictors, which account for all participating proteins in the interaction are less common but have the benefit of considering complementarity between specific proteins. Partner‐specific predictors use structures or sequences of two proteins that are assumed to interact in predicting the interaction interface for each protein.^18^ A partner‐specific approach allows the user to consider complementarity, which plays a central role in molecular recognition. Proteins that promiscuously bind to multiple partners present a unique challenge for predicting interfaces. These multiple binding partners may all bind at the same site, or they may bind at multiple sites on the protein surface.^24^ While recent studies highlight the ability of current predictors to separate non‐binding from binding residues on individual proteins, these predictors fail to distinguish partner‐specific interaction sites resulting in cross‐prediction between sites.^19^

Currently, many partner‐specific approaches exist for predicting interactions. A majority of these methods use the primary sequence and homology searches to make predictions. PAIRpred utilizes a support vector machine classifier for predicting partner‐specific interaction interfaces.^25^ While this approach employs multiple features, the features included in the classifier are all based on solvent accessible surface area, which cannot account for proteins that undergo a dramatic conformational change during binding. Another partner‐specific tool is PPIPP. PPIPP uses a neural network trained on interacting pairs and has been shown to outperform partner‐unaware models.^26^ Similarly, HomPPI uses sequence‐homology based approaches to identify conserved regions between the partners.^27^ Both approaches only use sequence information and do not incorporate spatial data. Many recent approaches have attempted to use multiple sequence alignments to predict residues that coevolve between proteins through direct coupling analysis, mutual information, or a combination of the two and show improved prediction capabilities.^8^, ^28^, ^29^

One important challenge that remains for partner‐specific, structure‐based predictors is accounting for conformational changes that occur upon binding. The performance of these methods decreases with increasing conformational rearrangements and dynamics of the protein pairs upon binding.^26^ For this reason, we were interested in developing a reduced representation of protein structural data that does not explicitly consider shape complementarity and can make quick predictions that may be used in assembling larger protein complexes. Here, we developed and evaluated a protein shape reduction method (MorphProt) that predicts partner‐specific interaction interfaces by mapping properties of protein surfaces to a reduced representation and rapidly tests for complementary surface patches within these reduced geometric representations. MorphProt shows comparable predictive power to a number of more computationally intensive approaches and tolerance to structural rearrangements in the interaction partners.

## 2 MATERIALS AND METHODS

### 2.1 Benchmark set of protein‐protein interactions

To evaluate the quality of the interaction interface predictions from MorphProt, we used a benchmark set of known protein complexes. The benchmark data set for this method was version 5.0 of the widely used protein‐protein interaction docking benchmarks.^30^ This benchmark set provides a large library of 230 Protein Data Bank^31^ (PDB) files for non‐redundant complexes with varying rigidity, as well as enzyme‐containing and antibody‐antigen complexes. From this set, we extracted 172 complexes (Supporting Information). Those complexes that were not included either had incomplete structures, creating an error in the PQR calculation or had more than two subunit chains (excluding antibody complexes).

In addition to the protein docking benchmark 5.0, we used the protein docking gold standard, the Critical Assessment of PRedicted Interactions (CAPRI) score set.^32^ CAPRI provides an expanded benchmark data set for evaluating scoring functions, which includes 13 published CAPRI targets. All predictions were made on unbound structures and were validated against the bound structures.

### 2.2 Calculated properties of surfaces

The properties that were used in these analyses were charge, hydrophobicity, and evolutionary rate. The atomic charge was calculated using PDB2PQR.^33^ PDB2PQR begins by rebuilding missing non‐hydrogen atoms using standard amino acid topologies in conjunction with the existing atomic coordinates to determine new positions for the missing atoms. Next, hydrogen atoms are added and positioned to optimize the global hydrogen‐bonding network. Finally, PDB2PQR assigns atomic charges and radii based on the AMBER force field. Here, The PDB2PQR program was run using the Opal server.

The Wimley‐White hydrophobicity values^34^ were used in determining residue hydrophobicity. These values are semi‐empirical and based on the transfer of free energies of polypeptides that show how favorable an amino acid is in a hydrophobic environment. Each atom in the atomic structure was assigned a hydrophobicity value based on the amino acid it was representing.

Finally, the evolutionary rates were obtained from the ConSurf Database.^35^ This database contains information regarding pre‐calculated evolutionary conservation scores. The evolutionary rates stored in the database are calculated using the Rate4Site algorithm.^36^ This method evaluates evolutionary rates using a maximum likelihood estimate assuming a stochastic process. Based on this, amino acid replacement probabilities were computed for each branch of the phylogenetic tree. The tree is then used to cluster closely related sequences and find a consensus sequence for each cluster. The consensus sequences are then compared, and each position may be described as variable or conserved. The frequencies are renormalized to represent a number between 1 and 9. Finally, each of the properties described was stored in the surface of the protein structure as part of the appropriate atomic coordinate.

### 2.3 Protein shape reduction

To reduce the dimensionality of the intricacies of protein shape, we performed a shape reduction of the 3D atomic structure into a simplified representation. To perform these calculations, we have created a Python library, MorphProt. The input for these calculations is a PDB file (either an atomic structure or homology model), a PQR file, and a conservation file produced by Consurf^35^ (when considering evolutionary rate). MorphProt began by extracting the molecular surface using Michel Sanner's MSMS program,^37^ which uses a 1.4 Å diameter sphere to detect the solvent accessible surface area. Next, it calculated a residue depth for all of the amino acids in the protein sequence using the molecular surface. The residue depth was calculated using Biopython^38^ and was evaluated as the average depth of all atoms in a residue from the calculated surface. In MorphProt, amino acids were said to be contributing to the surface of the protein if their residue depth (calculated as the average depth across all atoms in the residue) was less than 5 Å from the calculated accessible surface. We include this additional 5 Å from the accessible surface to account for any subsurface binding properties that may be missed in the accessibility calculation. MorphProt then extracted the 3D coordinates for all of the atoms that satisfy these surface constraints.

After the atomic coordinates of the surface are extracted, MorphProt took the maximum and minimum for each *x*, *y*, and *z* coordinate as 6 biased start centroids, *k* = 6. MorphProt uses SKLearn^39^ to perform a K‐means clustering. It projected each of the clusters onto a 2D surface proportional to the size of the cluster. Next, it binned each 2D projection into 5 Å by 5 Å boxes, forming a grid. Note that MorphProt allows for a customizable bin size. For these experiments, 5 Å by 5 Å boxes were used, as increases in bin size would lead to decreases in resolution. For each binned box, MorphProt calculated the average value of each atomic property in the box, creating a 2D matrix of values. Here, each matrix represents one of six faces of the protein. Each of these numbers in the matrix may be mapped back to a location on the protein surface. To avoid interaction interfaces being split along an edge of each face, each protein is rotated 45° in the *x* and *y* direction and the *k*‐means is recalculated. We also test an initialization of the protein structures by taking the first three principal components, corresponding to the major axes, to orient the protein in an optimized start position.

### 2.4 Protein interaction interface prediction

We computed a 2D cross‐correlation, a common pattern recognition and image processing tool, to predict areas of the protein surface with maximum interaction between properties. The cross‐correlation was calculated using MorphProt. Because each protein is reduced to a total of six matrices, we calculated a total of 36 2D cross‐correlations for each pairwise interaction between the six faces of each protein for a given initialization. In addition, we sampled all 10° rotations to account, in an approximate fashion, for different orientations or positions of the initial protein structures.

We then calculated the sum of the top 10 scores for the unrotated and rotated initialization positions. The top score for interactions driven by evolutionary rate and hydrophobicity is the maximum score; however, because complementary charge interactions involve a pairing of negative and positive numbers, the top score is the minimum. The start position with the top score is used as the optimal start position. Predicted interfaces are calculated independently for each of the properties. However, to determine the most likely interface, we determine consistency for the top 10 interfaces predicted for each property. If each of the top 10 interface predictions is a different subset of the same two faces (matrices), then this can be considered a consistent prediction and is selected as the top interface. For antibody complexes, we took the top charge score due to the variable binding region that would not be captured with evolutionary rate or the hydrophobic interaction between the heavy and light chains.

After identifying the top score in the cross‐correlation matrix, we determine the position of the two matrices that produced the score. We use the position of the high score within the cross‐correlation matrix to identify the alignment of the two matrices and extract any overlapping regions between the two. Once the areas of the two matrices that are interacting are identified, we map this back to the protein structures themselves.

### 2.5 Evaluation of predicted protein interaction interfaces

To evaluate our predictions, we calculated a confusion matrix to classify predicted interface residues as true positives, false positives, false negatives, and true negatives based on the predicted and actual classes. We defined a residue to be on the interaction interface if any atom from the residue is within 5 Å of an atom from the protein it is in complex with. We then evaluated our confusion matrix where the precision, recall, accuracy, and *F*_1_ score are defined accordingly:

$$\text{Precision}=\frac{\mathrm{TP}}{\mathrm{TP}+\mathrm{FP}}$$

$$\text{Recall}=\frac{\mathrm{TP}}{\mathrm{TP}+\mathrm{FN}}$$

$$\text{Accuracy}=\frac{\mathrm{TP}+\mathrm{TN}}{\mathrm{TP}+\mathrm{TN}+\mathrm{FP}+\mathrm{FN}}$$

$$F_{1}=2\;\frac{\text{Precision}\times \text{Recall}}{\text{Precision}+\text{Recall}}$$

Additionally, we have integrated an extreme value calculation to validate the “uniqueness” of the atomic properties. This demonstrates that placement along the interface is not a random distribution of points but a clustering of some property contributing to an interface. To calculate this, we randomly shuffled the properties associated with each atom and recalculated scores. We repeated this shuffle and scoring 1000 times to generate a distribution. If the score was an extreme value in the distribution, then the score is statistically significant and represented a clustering of a property at that location.

### 2.6 Interface predictions from other tools

MorphProt was compared to four different structure‐based prediction tools: Promate 2,^40^ PredUS 2,^41^ PIER,^42^ and SPPIDER^43^ on the CAPRI score_set complexes. The predictions were all generated using the following servers. The Promate 2 predictions were run at: http://bioportal.weizmann.ac.il/promate/. The default configuration was used. The proteins that had no predicted interface atoms were assigned statistics of 0. The SPPIDER predictions were run at: http://sppider.cchmc.org, using the predict interface from unbound structures option. The tradeoff between sensitivity and specificity used was 0.5 (balanced). The PredUS 2 predictions were run at: https://bhapp.c2b2.columbia.edu/PredUs/index_omega.html, using all default settings. The PIER predictions were run using the server at: http://abagyan.ucsd.edu/PIER/. The suggested cutoff score of 30 was used to predict the interface residues.

### 2.7 Simulation of structural distortion by normal mode analysis

To simulate structural distortion in the crystal structures from the test set we used elNémo^44^, a normal mode analysis. elNémo predicts the possible movements of a macromolecule through low‐frequency normal modes. The l and r unbound subunits of PDBID: 1FQJ, 1NZ8, 1US7, 2GTP, and 3CPH from the protein‐protein interaction docking benchmark were used. All default parameters were kept except for DQMIN and DQMAX, which were adjusted to 100 and 300, respectively, to allow more extreme distortion. Selected normal modes and PDBs can be found in the Supporting Information. Modes were selected based on large Cα‐RMSD from the wild‐type structure.

## 3 RESULTS

We wished to test if a highly simplified geometric representation of a 3D protein surface embedded with properties was sufficient to predict protein‐protein interactions, while being tolerant of possible molecular motions relevant to the interaction. We wanted to consider protein surface properties and how opposing surfaces complement each other when forming an interface, largely independent of protein shape. For this reason, we began with a reduction of the irregular shape of a protein by considering atoms near the surface of the protein, thus excluding the atoms that play a role in stabilizing the protein core and presumably make less of a contribution to protein‐protein interactions.

### 3.1 Simplified representation and interaction interface prediction

**FIGURE 1:**
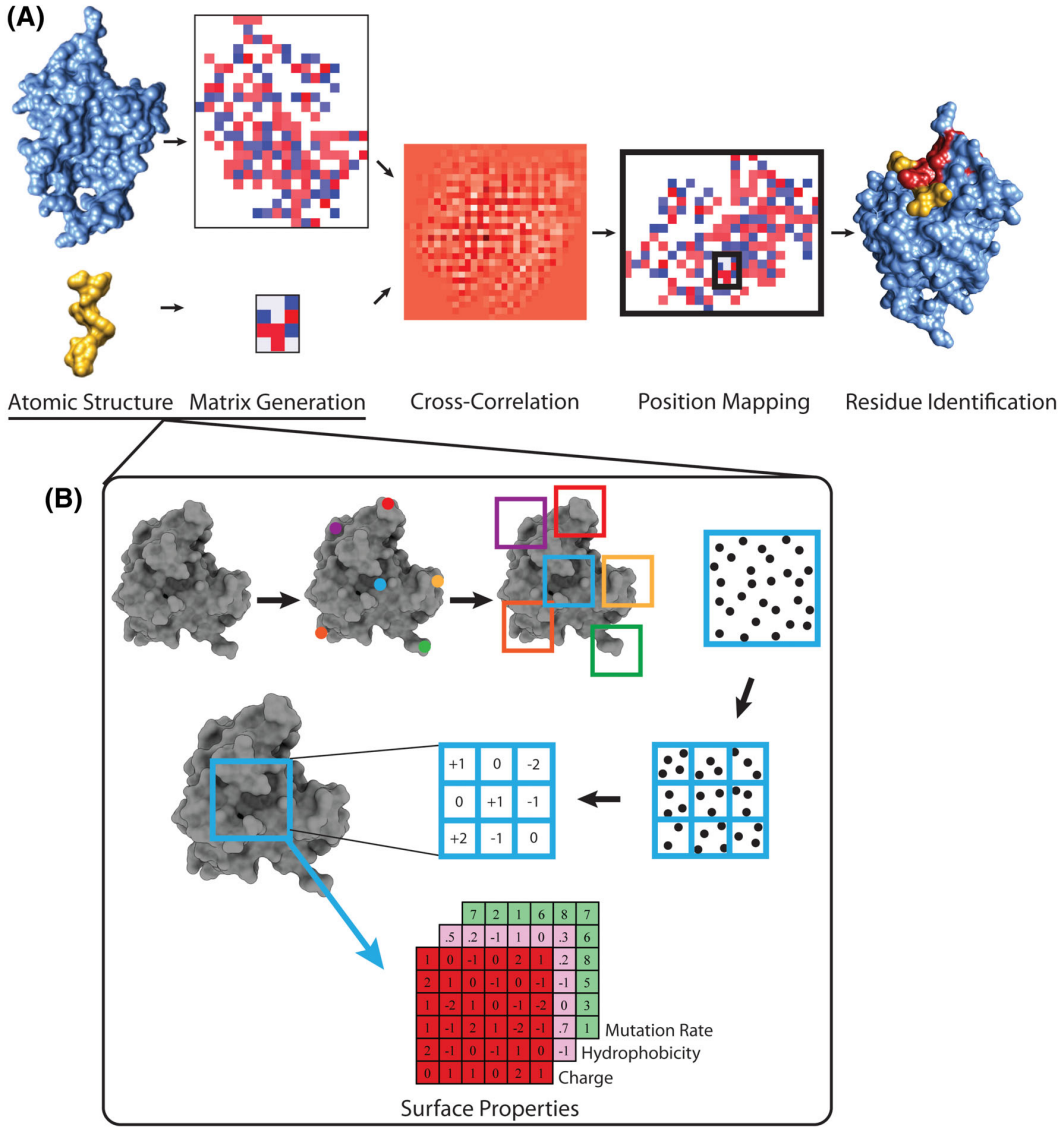
MorphProt pipeline for partner‐specific interaction interface prediction. A, The MorphProt interaction interface pipeline begins with atomic coordinates (PDB). Relevant surface properties are stored in these coordinates. The protein surface is converted into six matrices. A cross‐correlation is calculated between matrices of each protein to find the area of maximum interaction (max score). This is used to generate the position of the matrices to give the maximum, which are then mapped back to the surface of the protein. B, The atomic structure to property matrix is described in more detail. The surface of the structure is extracted, and a k‐means of the atomic coordinates is used to segment the surface into six patches. The patches are then projected into two dimensions. Each patch is then binned, and an average surface property is calculated

Our simplified representation is as follows: The solvent accessible surface of the protein is computed and reduced into a simplified geometric representation proportional to the size of the protein (Figure 1). The reduction retains an approximate representation of interface proportions. Recently, the idea of reducing proteins to simplified shapes has gained attention in structural searches.^45^ Our shape reduction uses a K‐means clustering algorithm to separate protein surface accessible amino acids into six distinct clusters, followed by a projection of the coordinates into two‐dimensions (2D) (Figure 1B) to represent the surfaces. Each atomic coordinate is described by its unique properties; charge, hydrophobicity, and evolutionary rate. These 2D coordinates are then binned into a grid based on the transformed atomic coordinate locations, and the average property value is calculated for each square of the grid. The result is a matrix of property values where the locations of the values within the matrix represent the neighbors of the atoms on the protein surface with minimal distortion.

These reduced protein surfaces are images, making them suitable for several image processing techniques. To build a partner‐specific predictor that considers surface property‐complementarity, we performed cross‐correlation of images from two partner proteins to find an area of maximum similarity between the two images by computing a dot product at each position after rotation and translation (Figure 1A). Cross‐correlations have already proven to be invaluable in various image processing techniques, including identifying single particles from electron microscopy data.^46^ Here, this approach was used to identify an area of maximum interaction by searching and calculating a complementarity score between properties in the matrix. Because our protein surfaces were reduced into six matrices, we cross‐correlated each matrix of one binding partner with each matrix of its partner and generated a score for each position of the 36 cross‐correlations. The highest scores represent the positions of each protein face where the maximum interaction occurs. The position of the matrices can be mapped back onto the protein surface that they represent. We designed a Python package called MorphProt to perform the shape reduction, cross‐correlation evaluations, and plot the predicted interface residues onto the atomic structure.

### 3.2 Detecting interaction interfaces with a known nature of interaction

**FIGURE 2:**
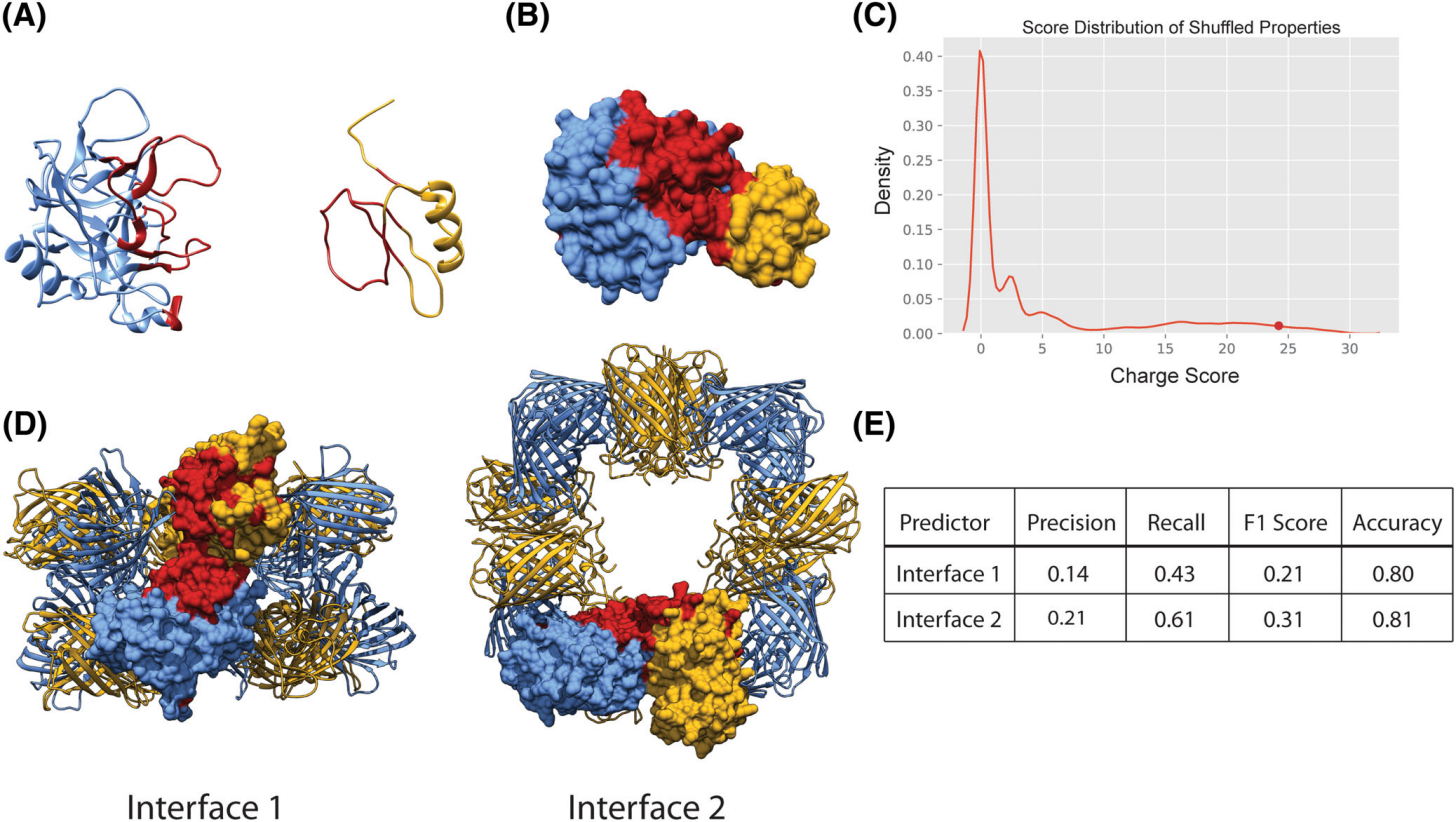
MorphProt interface predictions for known charged interfaces. A, The experimentally determined structure of the alpha‐chymotrypsin‐elgin c protein complex (PDB: 1ACB). The ligand or**l** (gold) interface residues were set to −1 and the receptor or**r** (light blue) interface residues were set to +1, while the remainder of the surface residues were assigned a charge of 0. B, The predicted interface (red) was mapped onto the protein complex. C, The cross‐correlation scores produced from 1000 shuffles of the engineered charge property across the surface of the protein. The point represents the top score from the prediction (*P*value: .007). D, MorphProt predicts interfaces of the Ceru+32/GFP‐17 super‐charged GFP protomer (PDB: 6MDR) between the alpha and beta subunits based on known charge‐based interactions. E, The statistics of the two interfaces are reported in the table [Color figure can be viewed at wileyonlinelibrary.com]

To address the concern of any distortion by the shape reduction, we demonstrated that interaction interfaces are still detectable with a proof‐of‐concept protein pair, the alpha‐chymotrypsin‐eglin c complex (PDB:1ACB) (Figure 2A‐C). We extracted the surface of each protein in the complex and set the charge property to 0 at all positions with the exception of the true interface. We defined the true interface as all atoms from one protein that are within 5 Å of an atom of the other protein in the complex. The atoms on the true interface of alpha‐chymotrypsin were assigned a charge of +1, and those on the true interface of eglin c were assigned a charge of −1. We then performed our shape reduction and cross‐correlation analysis using MorphProt. The top 10 interaction scores were all between the same two protein faces, which cluster along the true interface. This indicates that despite any distortion that occurs from our reduced representation of the protein surface, MorphProt was still able to identify the area of complementarity between the two surfaces. In addition, when the surface properties were shuffled, the true location of the property was identified as an extreme value (*P* value: .007). These results further support the notion that the shape reduction does not cause significant distortions, and cross‐correlation can be used to find the true interface of complementary properties. We then extended this approach to a more complicated scenario of a protein complex with known interaction basis.

Of primary interest for biological processes, is the assembly of large macromolecular machines. Using MorphProt, we explored the assembly of a large protein complex by examining our recently published Ceru+32/GFP‐17 protomer structure,^47^ a synthetically engineered supercharged GFP 16‐mer (Figure 2D,E). These proteins were engineered to have oppositely charged variants of the normally monomeric green fluorescent proteins (GFP), which resulted in the assembly of a large, ordered macromolecular structure. Because the structure is known to form charge‐based interactions, it served as an effective test for the ability of MorphProt to predict partner‐specific interactions within a large macromolecular complex where subunits have multiple interaction interfaces. The input for MorphProt was the *α* and *β* supercharged subunits. The top 10 scores accurately predicted two of the charge‐based interfaces between subunits. To further show that the engineered, supercharged GFP produces new charge‐based interfaces, we performed our shuffle analysis on wild‐type GFP to produce 396 000 possible scores and arrangements of the wild‐type residues. The scores from the supercharged GFP fell in the upper tail of the distribution of scores (Figure S1).

### 3.3 Evaluating MorphProt on a benchmark protein interaction set

**FIGURE 3:**
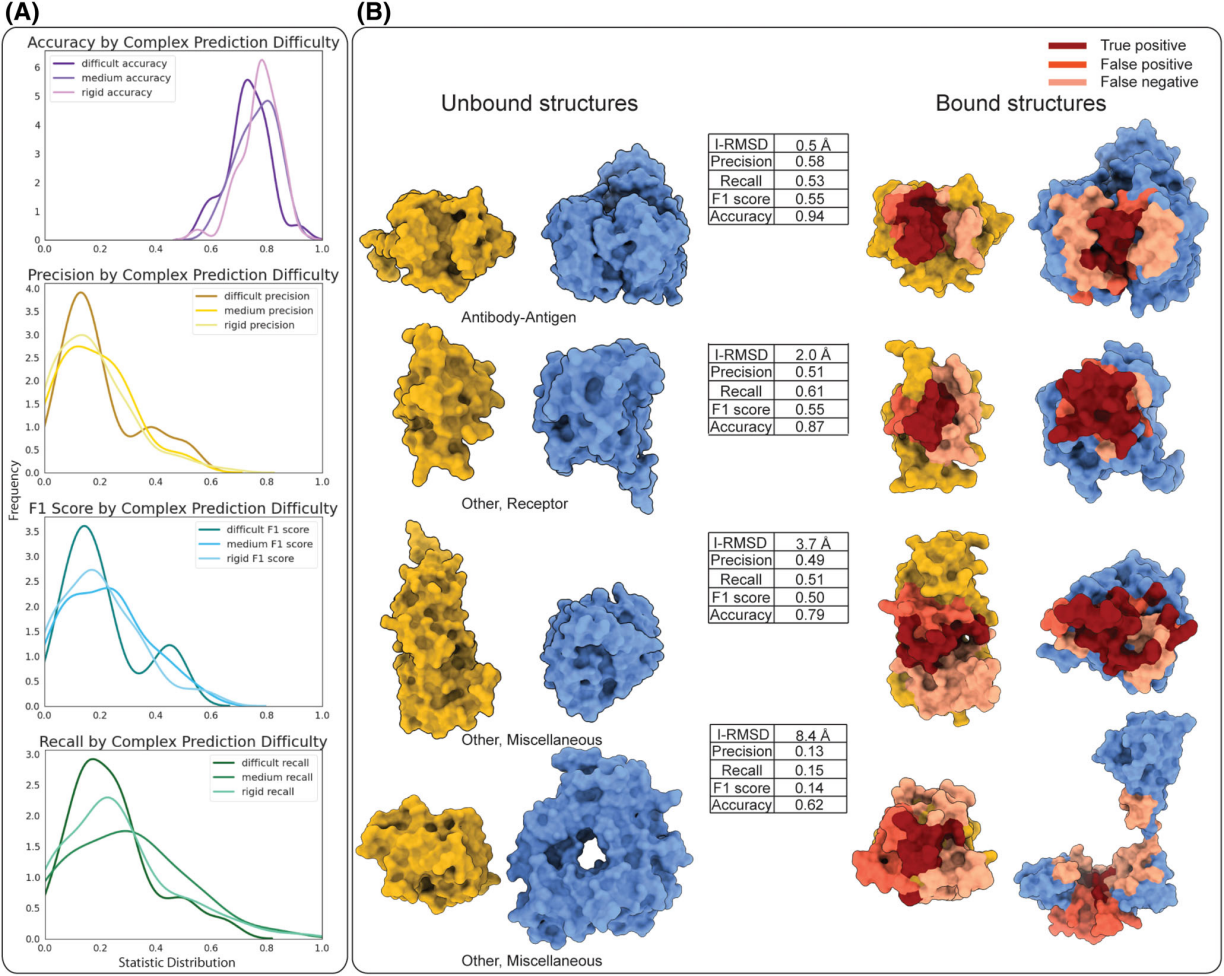
MorphProt predictions for the protein docking benchmark version 5. The protein docking benchmark groups protein complexes based on the difficulty in predicting the interaction interface or docking conformation. MorphProt predictions are made for the unbound structures and evaluated on the bound structures. A, The distribution of precision, recall, *F*_1_score, and accuracy for protein complexes in each difficulty group. B, The unbound structures for representative proteins from each of the difficulty groups are shown on the left. The bound protein structures with the predicted interface are colored in red. In each table, the type of interaction and I‐RMSD is reported from the protein docking benchmark version 5. The bottom complex has the largest conformational change upon binding from the difficult complex group [Color figure can be viewed at wileyonlinelibrary.com]

Because many times the properties contributing to the interaction interface remain elusive, we tested the MorphProt prediction on a combined benchmark set^30^, ^32^ of 185 protein complexes of varying interaction type. Of the protein complexes tested, 172 came from the docking benchmark version 5,^30^ which includes various types of interactions including antibody‐antigen, enzyme‐substrate, and enzyme‐receptor (Table S1). These complexes are also categorized by the difficulty to predict the interaction interface based on I‐RMSD (RMSD of the interface). Using our validation scheme, we have analyzed the ability of MorphProt to predict the interaction interface of the protein pairs for each difficulty group (Figure 3A,B). The average *F*_1_ score, a weighted combination of precision and recall, for the rigid, medium, and difficult complexes was 0.2, 0.22, and 0.21, respectively. The accuracy of the MorphProt prediction of the rigid, medium, and difficult complexes was 0.78, 0.76, and 0.73, respectively. We show that MorphProt's ability to predict interaction interfaces is fairly robust against structural changes upon binding with little variability between the statistics of each difficulty group (Figure 3A,B). The most severe conformational change had an I‐RMSD of 8.4 Å between the bound and unbound structures, resulting in the receptor protein opening to bind with the ligand. Despite this large conformational change, MorphProt was still able to predict some of the interaction interface (Figure 3B).

We also classified these predictions according to the CAPRI criterion of high, medium, acceptable, and incorrect ranking.^48^ Using this criterion, we found that 26 of the complexes ranked high, 35 ranked medium, 74 ranked acceptable, and 37 ranked incorrect. Our method is based on selecting the top, consistent interface as the prediction, but we were interested in exploring whether selecting the second interface for those predictions where recall <0.1 would improve our statistics (Figure S2). This approach led to better summary statistics with the new average *F*_1_ score of 0.22 (up from 0.21) and the accuracy 0.77 (up from 0.74). In addition, the new CAPRI criterion ranking was 30 high, 41 medium, 81 acceptable, and 20 incorrect.

In addition to the protein docking benchmark set, we tested MorphProt's performance on the 13 of the CAPRI targets from the score_set benchmark^32^ (Table 1). To determine whether a normalized start position would improve the clustering and predictions, we used a PCA to orient each protein so that the longest principal components were on each axis. When compared to MorphProt without PCA, we see a boost in both precision and recall. When compared to four other structure‐based interaction interface predictors (Promate 2, PredUS 2, PIER, and SPPIDER), we see that our reduced representation performs favorably on all statistical parameters (Table 1).

**TABLE 1:** Comparison of MorphProt with four other structure‐based interface predictors

Predictor	Precision	Recall	*F*_1_ Score	Accuracy
MorphProt	0.20	0.25	0.22	0.74
MorphProt PCA	0.23	0.26	0.24	0.74
SPPIDER	0.23	0.36	0.26	0.73
Promate 2	0.20	0.04	0.06	0.56
PredUS 2	0.40	0.32	0.34	0.82
PIER	0.42	0.12	0.17	0.84

*Notes:* The average prediction statistics across the Capri Score‐set from MorphProt, MorphProt with PCA, SPPIDER, Promate 2, PredUS2, and PIER are shown. All statistics were calculated in the same manner. An interface residue is any residue within 5 Å of a heavy atom from the other protein in the complex. All predictions were made on unbound structures and validated on the bound structures.

### 3.4 Interaction interface prediction despite structural distortion

**FIGURE 4:**
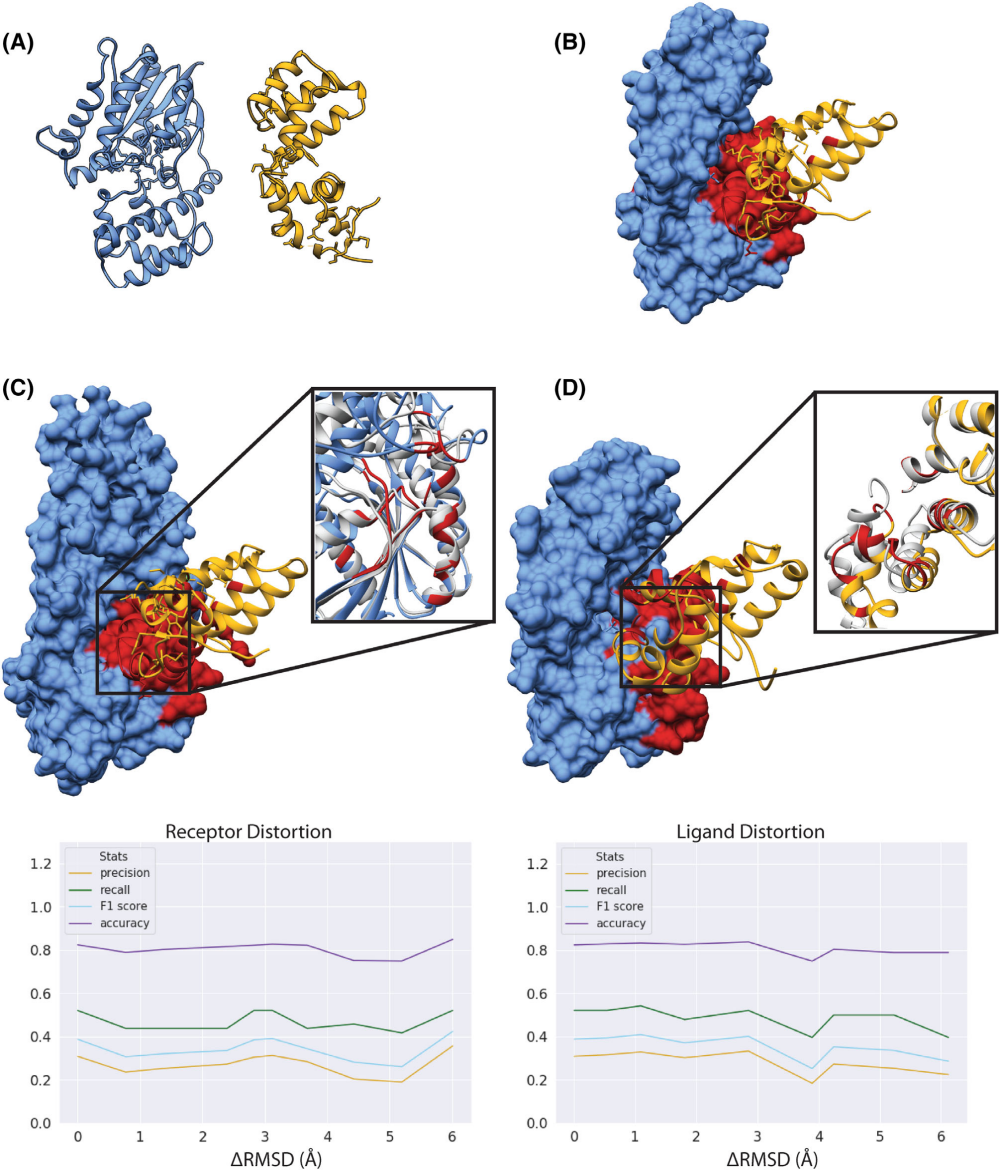
MorphProt can predict interaction interfaces despite structural distortion. A, Unbound structure of Gnai and RGS9 (PDB: 1FQJ). The ligand and receptor are depicted in gold and blue, respectively. The interface is predicted using evolutionary rate. B, The predicted interface is colored red on the bound structure. C, The receptor and ligand were distorted using elNémo normal mode analysis. The receptor was distorted up to ~6 Å (Cα‐RMSD) while ligand was held constant. The zoom‐in depicts the native structure (gray) superimposed onto the ~6 Å distorted structure to show the change in position of residues on the interface (top). Precision, recall, *F*_1_ score, and accuracy were plotted against Cα‐RMSD showing that for the distorted receptor there was no linear correlation between Cα‐RMSD (*R* ^2^_acc_ and *R* ^2^_*F*1_ < .02) (bottom). D, The same was done for the ligand while the receptor was held constant. Here, precision, recall, *F*_1_score, and accuracy showed a slight linear correlation with the Cα‐RMSD (*R* ^2^_acc_ = .44 and *R* ^2^_*F*1_ = .52) [Color figure can be viewed at wileyonlinelibrary.com]

Finally, we wanted to test the performance of our interface predictor against the uncertainty that may arise from structural models produced by homology modeling or lower resolution structure building methods. In both experimental and computational structural biology, there can occasionally be uncertainty regarding the exact position of the side chains and backbone of the model. By distorting one of our test proteins that produced a strong evolutionary rate interface prediction, we showed that our predictions remain robust even considering a structure that is distorted by up to ~6 Å Cα‐RMSD. The crystal structures of the unbound Gnai and RGS9 (PDB: 1FQJ) were distorted using normal mode analysis (Figure 4A,B). We used elNémo^44^ to compute the low‐frequency normal modes of each of the structures in the complex. In the analysis, one of the subunits (receptor or ligand) was held constant, while the interface was predicted at different Cα‐RMSD distortions of the other subunit (receptor or ligand). Despite different configurations of the protein backbone, we were still able to predict the interface based on the generalized property complementarity for a given section of the protein structure. We show the predicted interface after structural distortion of both the receptor and ligand (Figure 4C,D). At different points in the receptor distortion, we see some slight changes in the prediction summary statistics but no linear correlation (*R* ^2^ < .02). However, the ligand distortion shows a slight linear correlation (*R* ^2^ < .52), with the prediction statistics decreasing as the Cα‐RMSD of the ligand with the native ligand increases. This process was repeated for four additional complexes (eight structures) with similar results (Figure S3).

## 4 DISCUSSION

Here, we have demonstrated that by using shape reduction to normalize the highly variable 3D protein structure to a simplified geometric representation, we are able to store layers of information on a 2D representation of a protein surface while preserving atomic neighborhoods. The resulting matrix of values contains the location of surface properties and their proximity to other values and is a direct representation of the spatial coordinates of the 3D atomic structure. We showed that converting the surface properties to an image allows us to identify areas of maximum interaction of surface properties between two proteins via a partner‐specific approach. In addition, MorphProt has the ability to construct large macromolecular assemblies through detecting multiple partner‐specific interfaces.

While primary sequences provide information regarding amino acid identity and adjacent residues, it can be difficult to precisely determine from sequence alone which residues reside on the surface of a protein and their relation to each other in its 3D structure. Structure‐based approaches allow us to extract and investigate surface properties, providing a useful first step for interface prediction, as the spatial position of residues is essential for macromolecular recognition.^23^ Many machine learning interaction interface predictors exist and use structure, but the only information stored in feature vectors is statistical information for the surface patches and not the spatial arrangement of the residues.^23^ In addition to the lack of information regarding residue neighborhoods, many of the structure‐based approaches are not equipped to handle dramatic conformational changes upon binding.^49^ We have addressed these limitations of previous methods through our shape reduction by treating the protein surface as a simple 2D matrix, where the location of a value within the matrix is a representation of the location of that value on the protein surface. This novel surface‐patch approach turns out to be incredibly powerful in identifying the areas of maximum interaction between structures of interacting pairs.

In our approach, patch size is not predetermined; instead, it is dependent on the size of the proteins being tested and the size of overlap between protein faces for each score calculation. Traditional approaches for identifying a surface patch result in fairly uniform patch sizes.^22^ Our method tests surface patches over a number of different sizes and arrangements because the patches are determined by the position of the cross‐correlation. The first patch tested is the corner of two matrices and expands as the calculation continues, and the patches are both rotated and adjusted in size. The result is a sample of various patches and orientations, which can be used to identify the area of maximum interaction between the pair of proteins.

Because our interfaces are defined by a continuous patch of residues and not several interacting residues, it is worth investigating the best method of defining a true interface. In our evaluations, we focused on the CAPRI standard, which defines an interface residue as containing a heavy atom within 5 Å of a heavy atom of the other protein in the bound structure. However, because we are predicting whole interface patches, we have a large number of false positive predictions (residues on the outside of the patch or in concave sections of the surface) that may not be within the 5 Å threshold. By reevaluating our CAPRI predictions using a more forgiving 10 Å true interface threshold, we see the average precision shift from 0.20 to 0.42 (Figure S4). It is important to note that this new threshold leads to an increase in false negative predictions, resulting in a decrease in recall and accuracy. The *F*_1_ score, which combines both precision and recall, went from an average of 0.22 to 0.28. The evaluation criterion is an important consideration and is dependent on the intended use of the prediction.

In our comparison of MorphProt and MorphProt PCA with four other structural predictions, we show that the MorphProt methods are comparable to available methods. MorphProt, however, differs greatly from these methods in its approach. The reduced representation removes emphasis from the shape and shifts it to surface properties and their location relative to each other. Because of this, no information is required aside from the protein structure to generate a prediction. The other programs require some sort of *a priori* information, either a template that resembles the protein being searched or a training set where a similar protein is represented. This limits these methods from making predictions for proteins that are not well represented experimentally.

In most structure‐based interaction interface predictors, an interface is identified based on features of a given area on one of the protein surfaces, ignoring properties of a partner when determining how they best fit together. A partner‐specific predictor uses information regarding both proteins of interest. It has been shown that prediction methods that do not employ a partner‐specific approach have lower reliability in predicting transient binding sites,^50^, ^51^ whereas a partner‐specific approach can identify locations that are highly conserved for transient protein‐protein interactions.^27^ A significant advantage of using a partner‐specific predictor is its ability to find specific surface areas that form interactions with different partners. One significant challenge of many partner‐specific predictors is their use of unbound protein structures to search for interaction interfaces.^18^ In many biological processes, proteins undergo a dramatic conformational change upon binding, which complicates predicting an interface based on unbound structures. We have demonstrated that using a reduced surface representation of a protein in combination with stored information of highly predictive properties, we can make partner‐specific interface predictions for unbound proteins, including those that undergo at least moderate structural rearrangements, an important feature for building multi‐protein assemblies.

Furthermore, we showed that despite introduced structural distortion, we are still able to predict interfaces based on complementarity. This is increasingly important for predicting interaction interfaces with the widespread use of homology models and lower‐resolution structures. Here, greater weight is put on the neighborhoods of properties on the surface rather than their exact location. The ability to predict the interface for homology models is significant for assembling macromolecular complexes where little is known regarding the structure of the individual subunits. Theoretically, one could produce models for the subunits and then arrange them according to their interaction interfaces to predict the structure for large assemblies. Such analyses would also benefit from protein docking following the interface prediction to improve positioning.

While discrepancies between interface prediction and protein docking occur often, the techniques are effective when used in conjunction with one another. Protein‐protein docking is a partner‐specific technique that is highly dependent on shape complementarity and energetics.^23^ One of the limitations of protein‐protein docking is the large sample size that must be tested and then scored by an energy function to produce a prediction of the arrangement of two proteins. The number of arrangements would be drastically reduced by using an interface prediction as a preliminary step before docking. Previous studies showed that using a partner‐specific, homology‐based interface prediction prior to docking significantly improves the scoring of the docked proteins.^52^ Notably, the HADDOCK server allows for the incorporation of a predicted interaction interface, however, this interface is computed from a single protein rather than a partner‐specific interface.^53^ Incorporating our interface prediction into a protein‐protein docking pipeline would increase computational efficiency because it is independent of shape complementarity and energetics.

Another significant application of partner‐specific interaction interface predictors is the screening of small molecule inhibitors or drugs. These often interact via transient interactions,^23^ making predicting transient interactions imperative. Small molecules that interact with protein‐protein interfaces and alter these interactions have demonstrated to be effective drugs and the prediction of these interfaces could be useful in finding potential targets.^54^ This poses a challenge because many protein interfaces have been described as large, featureless surfaces that lack obvious binding pockets.^55^ Because our method reduces the protein surface to essentially the same, we would likely be able to make more accurate predictions using physicochemical properties stored on the surface of the protein. Furthermore, predictions and scores for small molecule inhibitors or drugs could be optimized by understanding the area of interaction produced by our method.

## 5 CONCLUSIONS

To address the inherent variability of protein shape, conformational changes, and structural approximations while reducing computation time, we were interested in determining if a simplified geometry retains enough spatial information to predict interaction interfaces based on complementary properties. Specifically, our aim was to develop a pipeline that was robust to molecular motions while gaining computational power to assemble larger multimeric protein complexes. Using MorphProt, we performed a shape reduction of the accessible surface of a protein into a reduced surface representation. This reduced representation allows for easy storage of intrinsic properties of the protein such as hydrophobicity, charge, and evolutionary rate to be embedded within each surface image. The result is a quantitative description of these properties across a protein surface enabling image processing techniques to identify complementarity between the properties of two interacting protein surfaces. We show this method can be useful when one of the above properties is the driving force of the interaction. MorphProt was able to predict interaction interfaces for the unbound CAPRI targets and the protein‐protein interaction benchmark complexes with comparable results to a number of other predictors. Additionally, MorphProt was able to predict interfaces for a large 16‐subunit oligomer, proteins with multiple binding sites, and crystal structures that have been distorted by up to ~6 Å Cα‐RMSD to mimic models built from lower resolution density maps or imperfect homology models. Our algorithm could be integrated into platforms that aim to assemble complicated protein architectures.

## CONFLICT OF INTEREST

The authors declare no conflict of interest.

6

### PEER REVIEW

The peer review history for this article is available at https://publons.com/publon/10.1002/prot.26020.

## Supporting information


**Appendix** **S1.** Supporting Information.

**Figure S1** Score distribution of shuffled wild‐type GFP charge scores. The shuffling method was applied to a wild‐type GFP molecule paired with another wild‐type GFP molecule to show all of the possible scores the two proteins could attain. The charge score was multiplied by ^−1^ to produce positive values here. The scores for the two interfaces predicted in Figure 2D,E are shown with red dots. Both fall in the upper tail of the distribution, showing the engineered GFP scores significantly higher than scores that may be attained with the wild‐type GFP.
**Figure S2**: Considering other consistent predictions improves statistics. The second predicted interface was selected for predictions with precision <0.1 in the protein docking benchmark version 5, shifting the all statistics to the right. This suggests that considering all consistent interfaces might improve the prediction.
**Figure S3**: Interface predictions on additional normal mode analyses. Normal mode analysis was performed on 4 additional complexes, providing 8 more examples of interface predictions of distorted structures. The four complexes were taken from the benchmark set and were distorted up to 10 Å ΔCɑ‐RMSD from the native structure using normal mode analysis. Despite these alternative structures, MorphProt was still able to predict the interaction interface.
**Figure S4**: Statistical shift with change in interface definition. Changing the definition of an interface atom changes the distribution of precision, recall, F1 score, and accuracy. Because MorphProt selects all atoms predicted to be on an interface, not simply those side chains directly interacting with another protein, changing the definition of interface residues from being within 5 Å to 10 Å of a heavy atom of the other protein results in increased precision and F1 score, and decreased recall and accuracy.
**Table S1**: MorphProt predictions for different types of interactions. The table shows average precision, recall, F1 score, and accuracy for the different types of interactions in the protein docking benchmark version 5: antibody‐antigen, antigen‐bound antibody, enzymeinhibitor, enzyme‐regulatory/accessory, enzyme‐substrate, G‐protein, receptor, and miscellaneous.
**Table S2**: Normal modes selected for MorphProt interface prediction. The normal modes that were selected for each PDB to analyze the effect of molecular motions on the interface prediction.
