# Supplementary material for: Simplified geometric representations of protein structures identify complementary interaction interfaces
Source: Proteins. 2020 Nov 11;89(3):348–60. doi: 10.1002/prot.26020 (PMC7855953; doi:10.1002/prot.26020)
Supplement: Supplementary file 2 — Figure S1 Score distribution of shuffled wild‐type GFP charge scores. The shuffling method was applied to a wild‐type GFP molecule paired with another wild‐type GFP molecule to show all of the possible scores the two proteins could attain. The charge score was multiplied by −1 to produce positive values here. The scores for the two interfaces predicted in Figure 2D,E are shown with red dots. Both fall in the upper tail of the distribution, showing the engineered GFP scores significantly higher than scores that may be attained with the wild‐type GFP. Figure S2: Considering other consistent predictions improves statistics. The second predicted interface was selected for predictions with precision <0.1 in the protein docking benchmark version 5, shifting the all statistics to the right. This suggests that considering all consistent interfaces might improve the prediction. Figure S3: Interface predictions on additional normal mode analyses. Normal mode analysis was performed on 4 additional complexes, providing 8 more examples of interface predictions of distorted structures. The four complexes were taken from the benchmark set and were distorted up to 10 Å ΔCɑ‐RMSD from the native structure using normal mode analysis. Despite these alternative structures, MorphProt was still able to predict the interaction interface. Figure S4: Statistical shift with change in interface definition. Changing the definition of an interface atom changes the distribution of precision, recall, F1 score, and accuracy. Because MorphProt selects all atoms predicted to be on an interface, not simply those side chains directly interacting with another protein, changing the definition of interface residues from being within 5 Å to 10 Å of a heavy atom of the other protein results in increased precision and F1 score, and decreased recall and accuracy. Table S1: MorphProt predictions for different types of interactions. The table shows average precision, recall, F1 score, and accuracy for the diffe [file PROT-89-348-s002.pdf]

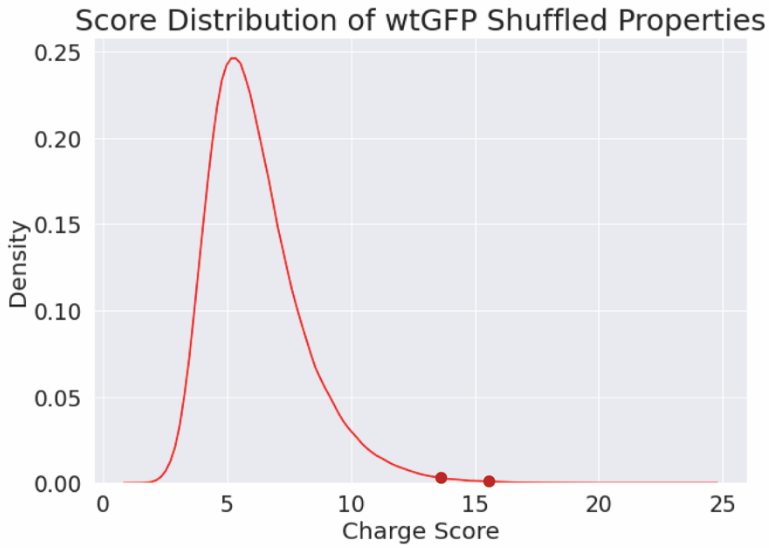

Supplementary Figure 1: Score distribution of shuffled wild-type GFP charge scores. The shuffling method was applied to a wild-type GFP molecule paired with another wild-type GFP molecule to show all of the possible scores the two proteins could attain. The charge score was multiplied by -1 to produce positive values here. The scores for the two interfaces predicted in **Figure 2d & e** are shown with red dots. Both fall in the upper tail of the distribution, showing the engineered GFP scores significantly higher than scores that may be attained with the wild-type GFP.

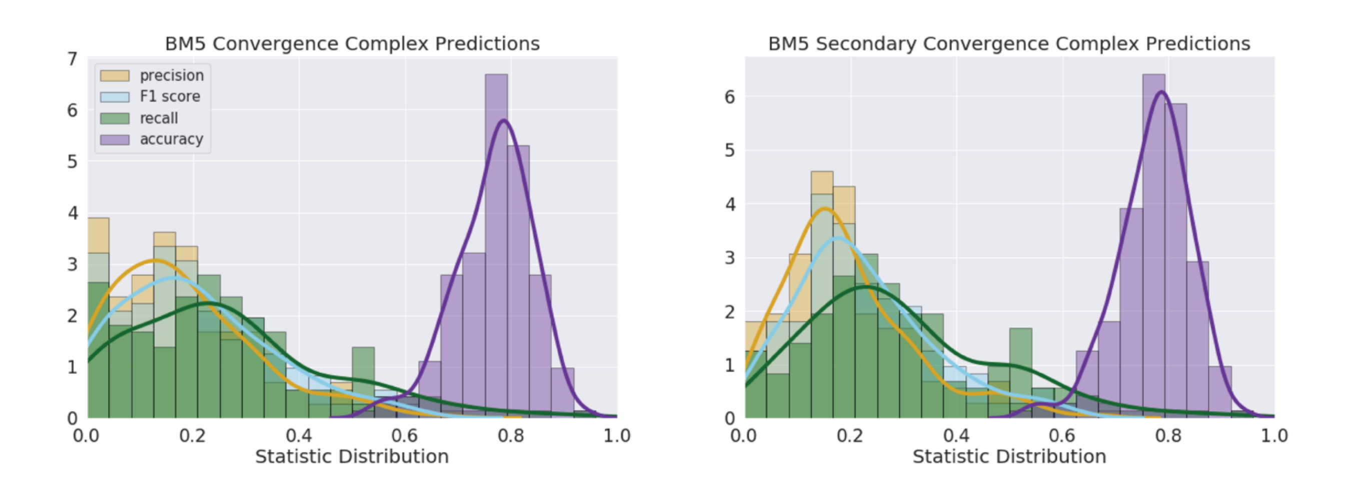

Supplementary Figure 2: Considering other consistent predictions improves statistics. The second predicted interface was selected for predictions with precision < 0.1 in the protein docking benchmark version 5, shifting the all statistics to the right. This suggests that considering all consistent interfaces might improve the prediction.

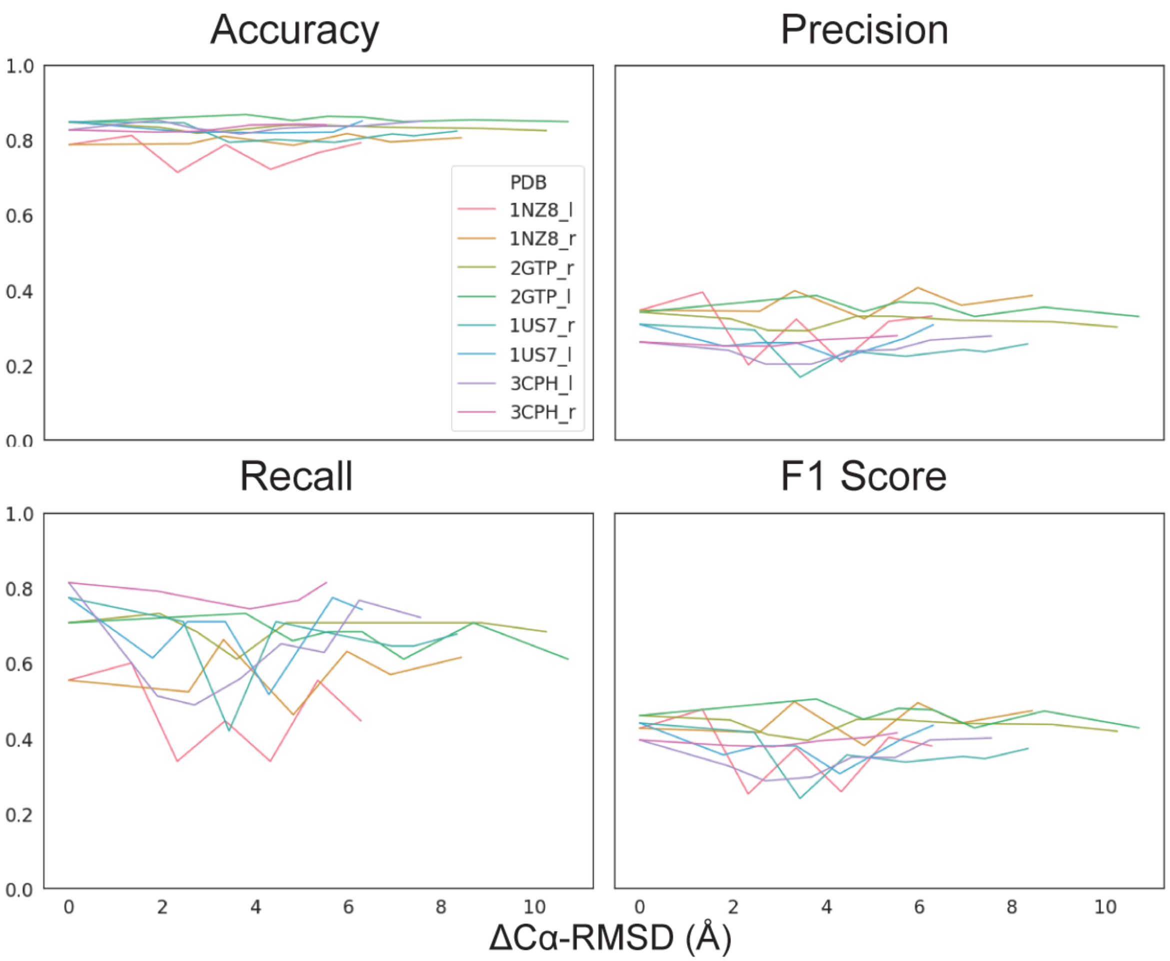

Supplementary Figure 3: Interface predictions on additional normal mode analyses. Normal mode analysis was performed on 4 additional complexes, providing 8 more examples of interface predictions of distorted structures. The four complexes were taken from the benchmark set and were distorted up to 10 Å ΔCα-RMSD from the native structure using normal mode analysis. Despite these alternative structures, MorphProt was still able to predict the interaction interface.

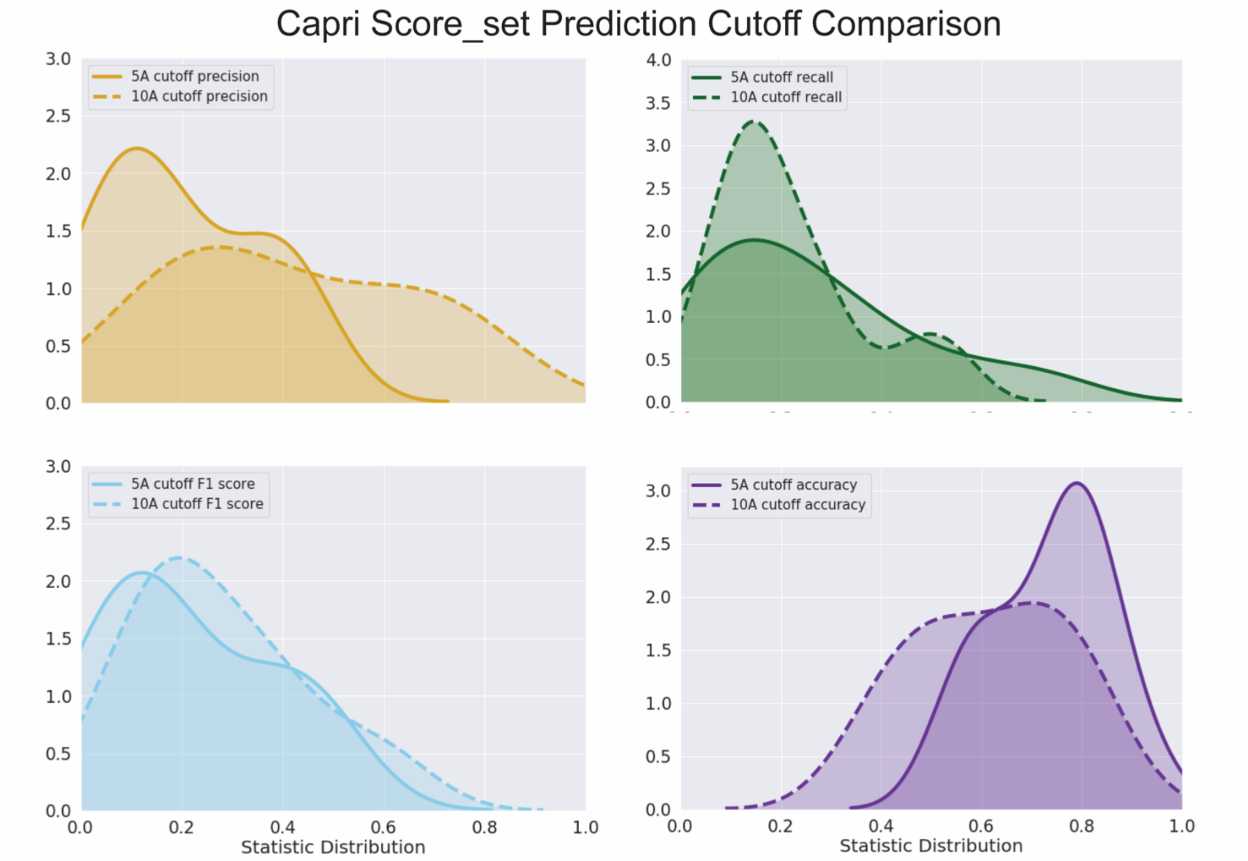

Predictor	Precision	Recall	F1 Score	Accuracy
MorphProt 5 Å	0.20	0.25	0.22	0.74
MorphProt 10 Å	0.42	0.22	0.28	0.62

Supplementary Figure 4: Statistical shift with change in interface definition. Changing the definition of an interface atom changes the distribution of precision, recall, F1 score, and accuracy. Because MorphProt selects all atoms predicted to be on an interface, not simply those side chains directly interacting with another protein, changing the definition of interface residues from being within 5 Å to 10 Å of a heavy atom of the other protein results in increased precision and F1 score, and decreased recall and accuracy.

Interaction Type	Precision	Recall	F1 Score	Accuracy
A	0.17	0.30	0.21	0.83
AB	0.13	0.29	0.17	0.81
ES	0.20	0.32	0.23	0.76
ER	0.26	0.38	0.30	0.78
EI	0.17	0.19	0.18	0.76
OG	0.23	0.40	0.28	0.78
OR	0.13	0.18	0.14	0.73
OX	0.17	0.22	0.18	0.74

A= Antibody-Antigen
AB= Antigen-Bound Antigen
ES= Enzyme-Substrate
ER= Enzyme-Receptor
EI= Enzyme-Inhibitor
OG= Other, G-protein
OR= Other, Receptor
OX= Other, Miscellaneous

Supplementary Table 1: MorphProt predictions for different types of interactions. The table shows average precision, recall, F1 score, and accuracy for the different types of interactions in the protein docking benchmark version 5: antibody-antigen, antigen-bound antibody, enzyme-inhibitor, enzyme-regulatory/accessory, enzyme-substrate, G-protein, receptor, and miscellaneous.

PDB	Normal Models
1FQJ_l	1, 4
1FQJ_r	1, 2
1NZ8_l	7, 8, 11
1NZ8_r	7, 8, 11
1US7_l	7, 11
1US7_r	7, 11
2GTP_l	8, 11
2GTP_r	7, 11
3CPH_l	7, 8, 11
3CPH_r	7, 8, 11

Supplementary Table 2: Normal modes selected for MorphProt interface prediction. The normal modes that were selected for each PDB to analyze the effect of molecular motions on the interface prediction.

Supplementary Methods

Pseudocode for MorphProt
The actual code can be found at: https://github.com/cmccaffe/MorphProt

Property selection
For charge:
If the sum of the top ten scores for the initial positions is less than the top ten scores of the rotated positions, we choose the initial positions. Otherwise, we choose the rotated positions.

For hydrophobicity and evolutionary rate:
If the sum of the top ten scores for the initial positions is greater than the top ten scores of the rotated positions, we choose the initial positions. Otherwise, we choose the rotated positions.

For each property (charge, hydrophobicity, evolutionary rate):
The occurrences of the most common face pair in the top ten scores is counted.

The max occurrences of the three properties is chosen. That is the property that is consistently giving a high score between the same two faces.

Between the top scoring faces chosen by the property selected above, we select the highest scoring position of the cross-correlation.

Matrix mapping
Face1 is the face of protein 1 that gave the high score.
Face2 is the face of protein 2 that gave the high score when cross-correlated with *face1*.
Score loc is the location in the cross correlation matrix that gave the highest score, *CnCm*.

Face1 is a *n1* by *m1* matrix and *face2* is a *n2* by *m2* matrix.

For the n interval for each face
If *n1* is greater than or equal to *n2*:
    If *Cn* is greater than or equal to *n1*:
        The *n-interval* of overlap for *face1* is (*Cn-n2*, *n1*)
        The *n-interval* of overlap for *face2* is (0, *n2-Cn+n1*)
    Else:
        If *Cn* is less than or equal to *n2*:
            The *n-interval* of overlap for *face1* is (0, *Cn*)
            The *n-interval* of overlap for *face2* is (*n2-Cn*, *n2*)
        Else:
            The *n-interval* of overlap for *face1* is (Cn-n2, Cn)
            The *n-interval* of overlap for *face2* is (0, *n2*)
Else:
    If *Cn* is greater than or equal to *n1*:
        If *Cn* is less than or equal to *n2*:

The *n-interva*l of overlap for *face1* is (0, *n1*)
The *n-interval* of overlap for *face2* is (*n2-Cn*, *n2-Cn+n1*)
Else:
The *n-interval* of overlap for *face1* is (*Cn-n2*, *n1*)
The *n-interval* of overlap for *face2* is (0, *n2-Cn+n1*)
Else:
The *n-interval* of overlap for *face1* is (0, *n2-Cn+n*1)
The *n-interval* of overlap for *face2* is (*n2-Cn*, *n2*)

For the m interval for each face
If *m1* is greater than or equal to *m2*:
If *Cm* is greater than or equal to *m1*:
The *m-interval* of overlap for *face1* is (*Cm-m2*, *m1*)
The *m-interval* of overlap for *face2* is (0, *m2-Cm+m1*)
Else:
If *Cm* is less than or equal to *m2*:
The *m-interval* of overlap for *face1* is (0, *Cm*)
The *m-interval* of overlap for *face2* is (*m2-Cm*, *m2*)
Else:
The *m-interval* of overlap for *face1* is (*Cm-m2*, *Cm*)
The *m-interval* of overlap for *face2* is (0, *m2*)
Else:
If *Cm* is greater than or equal to *m1*:
If *Cm* is equal to or less than *m2*:
The *m-interval* of overlap for *face1* is (0, *m1*)
The *m-interval* of overlap for *face2* is (*m2-Cm*, *m2-Cm+m1*)
Else:
The *m-interval* of overlap for *face1* is (*Cm-m2*, *m1*)
The *m-interval* of overlap for *face2* is (0, *m2-Cm+m1*)
Else:
The *m-interval* of overlap for *face1* is (0, *m2-Cm+m1*)
The *m-interval* of overlap for *face2* is (*m2-Cm*, *m2*)

Each n by m interval gives the area of *face1* and *face2* that is on the interaction interface.
